# A new argasid tick species (Acari: Argasidae) associated with the rock cavy, *Kerodon rupestris* Wied-Neuwied (Rodentia: Caviidae), in a semiarid region of Brazil

**DOI:** 10.1186/s13071-016-1796-7

**Published:** 2016-09-21

**Authors:** Marcelo B. Labruna, Santiago Nava, Arlei Marcili, Amalia R. M. Barbieri, Pablo H. Nunes, Mauricio C. Horta, José M. Venzal

**Affiliations:** 1 Departamento de Medicina Veterinária Preventiva e Saúde Animal, Faculdade de Medicina Veterinária e Zootecnia, Universidade de São Paulo, Av. Prof. Orlando Marques de Paiva 87, São Paulo, 05508-270 Brazil; 2 Instituto Nacional de Tecnología Agropecuaria, Estación Experimental Agropecuaria Rafaela and Consejo Nacional de Investigaciones Científicas y Técnicas, CC 22, CP 2300 Rafaela, Santa Fe Argentina; 3 Universidade de Santo Amaro, R. Prof. Enéas de Siqueira Neto 340, São Paulo, 04829-300 Brazil; 4 Instituto Latino-Americano de Ciências da Vida e da Natureza, Universidade Federal da Integração Latino-Americana, Av. Tarquínio Joslin dos Santos 1000, Foz do Iguaçu, Paraná 85870-901 Brazil; 5 Universidade Federal do Vale do São Francisco, Campus de Ciências Agrárias, Rodovia BR 407, Km 12, Lote 543 - Projeto de Irrigação Senador Nilo Coelho, s/n, Petrolina, Pernambuco 56300-990 Brazil; 6 Laboratorio de Vectores y Enfermedades Transmitidas, Facultad de Veterinaria, CENUR Litoral Norte, Salto, Universidad de la República, Rivera 1350, CP 50000 Salto, Uruguay

**Keywords:** *Ornithodoros rietcorreai*, *Kerodon rupestris*, Morphology, Mitochondrial 16S rDNA, ITS2

## Abstract

**Background:**

The rock cavy *Kerodon rupestris* (Wied-Neuwied, 1820) is a rodent species endemic to northeastern Brazil. Earlier studies have associated the argasid tick *Ornithodoros talaje* (Guérin-Méneville, 1849) with rocky cavy; however, a recent study proposed that *O. talaje* is not established in Brazil, where previous reports of this species were possibly misidentifications of closely related species, yet to be properly determined. Here, we describe a new species of *Ornithodoros* Koch, 1844 associated with rock cavies in northeastern Brazil*.*

**Methods:**

During 2012–2013, *Ornithodoros* ticks were collected from *K. rupestris* resting places in Paraíba State (PB) and Piauí State (PI), northeastern Brazil. These ticks were brought alive to the laboratory, and used to form two laboratory colonies (PB and PI ticks). Field-collected adults and laboratory-reared larvae were used for morphological description through light and scanning electron microscopy. DNA sequences of the mitochondrial 16S rRNA gene were generated from nymphal ticks and used to conduct phylogenetic analyses along with other *Ornithodoros* spp. sequences from GenBank. Reproductive compatibility of crosses between PB and PI adult ticks was evaluated, as well as analyses of hybrid ticks through larval morphology by a principal components analysis (PCA) and DNA sequences of the second internal transcribed spacer (ITS2) region from adult ticks.

**Results:**

Morphological analysis allowed recognizing these ticks as a new species, *Ornithodoros rietcorreai* n. sp. The larva of *O. rietcorreai* is distinct from those of other *Ornithodoros* spp. by the combination of the following character states: 14 pairs of dorsal setae, dorsal plate pyriform, hypostome with pointed apex and dental formula 3/3 anteriorly, 2/2 posteriorly, and anal valves with long and pointed leaf-shaped ends. There were a few larval morphological differences between PB and PI ticks*,* and their mitochondrial 16S rDNA sequences diverged by 3.3 %. On the other hand, cross-mating experiments showed that PB and PI ticks were reproductive compatible, indicating that they represent a single species. Analyses of ITS2 sequences and PCA corroborated this assumption.

**Conclusion:**

*Ornithodoros rietcorreai* is described as a new species associated with *K. rupestris* in Brazil, increasing the Brazilian tick fauna to 70 species.

## Background

The rock cavy, *Kerodon rupestris* (Wied-Neuwied, 1820)*,* is a rodent species endemic to the semiarid region (Caatinga biome) of northeastern Brazil. Adult rock cavies can weigh up to 1 kg, body length reaching up to 40 cm. They live in groups, inhabiting rocky areas with low, scrubby vegetation, typical of Caatinga hills, where they usually shelter in holes or crevices between rocks [1]. To the best of our knowledge, literature data about ticks infesting rock cavies under natural conditions have been restricted to a few earlier studies, which reported only two species on *K. rupestris*: the ixodid *Amblyomma parvum* Aragão, 1908 and the argasid *Ornithodoros talaje* (Guérin-Méneville, 1849) [2–4]. In addition, several collections of *O. talaje* from rock cavy resting places have been reported [2, 5, 6].

Previous reports of *O. talaje* in Brazil relied primarily on morphological examination of adult and nymphal ticks [2–6]. In the subgenus *Alectorobius*, species morphological discrimination with certainty is usually possible only through the larval stage [7–9]. After subsequent studies during the 1960s and 1970s, it is currently accepted that the nymphal and adult stages of most of the ‘New’ World *Ornithodoros* species (including *O. talaje*) belonging to this subgenus cannot be accurately separated through morphological analysis. In addition, recent studies proposed that *O. talaje* is not established in Brazil, where previous reports of this species were possibly misidentification of closely related species, further reported during the last decades or yet to be described [10, 11].

In this study, we report *Ornithodoros* ticks associated with rock cavies in northeastern Brazil, including areas where this host was previously reported to be infested by *O. talaje.* Our results indicate a new *Ornithodoros* species, which we describe for the first time.

## Methods

### Collection of ticks

On 10 September 2012, we collected 24 *Ornithodoros* ticks from crevices in a rocky hill inhabited by wild *K. rupestris* in São José de Espinharas Municipality (06°50’S, 37°24’W; elevation: 220 m), State of Paraíba (PB), northeastern Brazil. These ticks were brought alive to the laboratory, where they were used to form a laboratory colony, designated as PB ticks. On 22 January 2013, we collected 28 *Ornithodoros* ticks from crevices and the walls of a rocky hole inhabited by wild *K. rupestris* and bats (species not determined) in Jurema Municipality (08°52′53.7″S, 43°09′43.0″W; elevation 557 m), State of Piauí (PI), northeastern Brazil. Another sample of 10 ticks was collected from a rocky hole (08°53′27.4″S, 43°07′53.4″W; elevation 554 m), also inhabited by *K. rupestris* and bats (species not determined), 3 km distant from the first locality of Jurema. These ticks were brought alive to the laboratory, where they were used to form two laboratory colonies, designated as PI-1 and PI-2 ticks.

### Morphological analyses

Adult female ticks from two laboratory colonies (PB and PI-1) were fed on naïve hamsters in the laboratory and held in an incubator at 25 °C and a relative humidity of 80 %, where they laid egg masses that resulted in hatched larvae. Part of the unfed larvae (F_1_), when 15–20 day-old, where killed in hot water and preserved in 70 % ethanol for morphological analyses. For this purpose, unfed larvae from each colony were mounted in Hoyer’s medium to make semi-permanent slides and examined by light microscopy using a Nikon Eclipse E200 optical microscope for morphological analysis and morphometry.

For the description, a total of 67 morphological features were observed and/or measured using 10 larvae from each colony (PB and PI-1). Larval terminology and measurements follow Kohls et al. [7] with the modifications proposed by Estrada-Peña et al. [12] and Venzal et al. [10]. Measurements are given in millimeters, with the range followed by the mean in parentheses. Five PB larvae were prepared for scanning electron microscopy as previously described [13].

Five adults of each sex from each of PB and PI-I tick colonies were measured under a stereoscope microscope with the use of the Image-Pro Plus 5.1 program for analysis of images and morphometry, fitted to an Olympus SZX stereoscope microscope (Olympus Corporation, Tokyo, Japan). Two PB adults of each sex were prepared for scanning electron microscopy following previously described techniques [14]. Photomicrographs were taken with a Hitachi TM3000 scanning electron microscope (Hitachi High-Technologies Corporation, Tokyo, Japan).

The type-series of the tick species described in this study has been deposited in the following tick collections: United States National Tick Collection, Statesboro, GA, United States (USNMENT, CEN); “Coleção Nacional de Carrapatos”, University of São Paulo, SP, Brazil (CNC); Acari Collection of the Butantan Institute, University of São Paulo, SP, Brazil (IBSP); Department of Veterinary Parasitology, Faculty of Veterinary, Salto, Uruguay (DPVURU); Instituto Nacional de Tecnología Agropecuaria, Rafaela, Santa Fe, Argentina (INTA).

### Molecular and phylogenetic analyses

Two nymphs of each of the three tick colonies (PB, PI-1, and PI-2) were used for molecular analysis. For this purpose, each individual tick was subjected to DNA extraction with DNeasy Tissue Kit (Qiagen, Valencia, California), and processed by polymerase chain reaction (PCR) with the use of primers targeting a ≈ 460 bp fragment of the tick 16S rDNA mitochondrial gene, as previously described [15]. PCR products of the expected size were sequenced in an ABI automated sequencer (Applied Biosystems/Thermo Fisher Scientific, model ABI 3500 Genetic Analyzer, Foster City, California, USA) with the same primers used for PCR. The newly-generated sequences were compared to each other and submitted to BLAST analyses (www.ncbi.nlm.nih.gov/blast) to infer closest similarities available in GenBank.

The 16S rDNA mitochondrial gene partial sequences generated in this study were deposited in the GenBank database under accession numbers KX130781 and KX130782. These sequences were manually aligned using GeneDoc software (http://www.nrbsc.org/downloads/) with sequences previously determined for other argasid species available in GenBank, and also with sequences of *Ixodes holocyclus* Neumann, 1899 and *Ixodes uriae* White, 1852 (Ixodidae) that were used as a distant outgroup. The phylogenetic tree was inferred by the maximum parsimony method using PAUP version 4.0b10 [16] with 500 replicates of random addition taxa and tree bisection and reconnection branch swapping; all positions were given equal weight.

### Biological analyses and cross-mating assays

Part of the unfed larvae (F_1_) hatched from eggs laid by PB and PI-1 tick colonies were reared separately until the F_1_ adult stage in the laboratory. For this purpose, one tick-naïve hamster (*Mesocricetus auratus* Waterhouse, 1839) or vesper mouse [*Calomys callosus* (Rengger, 1830)] was used for feeding of each tick stage of each tick colony, except for N1 nymphs, which molted to N2 without feeding. Engorged N3 nymphs were held separately from each other by placing each tick inside a well of a 96-well plate covered by a plastic cover. Under this condition, the adult ticks (males and females) that emerged from N3 nymphs did not have the chance to copulate. While some N3 nymphs molted to N4 nymphs, only adult ticks that emerged from N3 nymphs were used for the cross-breeding assays.

Virgin, adult unfed F_1_ ticks were sorted in four plastic vials as follows: 5 PB males with 6 PB females in a vial (♂PB × ♀PB homologous cross); 8 PI-I males with 7 PI-I females in a vial (♂PI × ♀PI homologous cross); 5 PB males with 7 PI-I females (♂PB × ♀PI heterologous cross), and 8 PI-I males with 6 PB females (♂PI × ♀PB heterologous cross). These vials were left in the incubator for 14 days. After this period, adults of each cross were fed on a hamster; then, 3 to 4 couples from each cross were placed in different vials (1 couple per vial). When the female of each couple laid eggs, the hatched larvae (F_2_ ticks) were reared until the adult stage, representing F_2_ adult ticks that came from a single female. These F_2_ adult ticks (males and females) were fed on a hamster and held together in a vial to verify their fertility. In parallel, a sample of 3 F_1_ female ticks from either PB or PI colonies was always held without males (both before and after feeding on hamster) in order to verify if these ticks could reproduce by parthenogenesis.

### Morphological and molecular analyses of cross-mating ticks

Larvae (F_3_) produced by F_2_ adult ticks of all four crosses (♂PB × ♀PB; ♂PI × ♀PI; ♂PB × ♀PI; ♂PI × ♀PB) were killed in hot water, preserved in 70 % ethanol, and then mounted in Hoyer’s medium to make semi-permanent slides and examined by light microscopy for morphological and morphometric analyses, as described above for F_1_ larvae.

After the fertility tests, F_2_ adult ticks of all four crosses were subjected to DNA extraction as described above and processed by PCR targeting the second internal transcribed space (ITS2) of the tick ribosomal DNA. For this purpose, we used the primers Forward (5′-GGT GTG AAT TGC AGG ACA CAC TG-3′), corresponding to the 5.8S rDNA gene, and Reverse (5′-AGA TCA GGC GAG ACA ACC CGC-3′), corresponding to the 5’ end of the 28S rDNA gene. These primers were designed in this study to amplify a fragment of ≈ 550 bp of *Ornithodoros* ticks (unpublished data), which includes the complete ITS2 region (≈430 bp). PCR products of the expected sizes were cloned by using the TA Cloning kit (Invitrogen, Carlsbad, California, USA) following the manufacturer’s instructions. Plasmids containing the DNA inserts of the expected size were sequenced at least four times by using an ABI automated sequencer with M13 forward and M13 reverse sequencing primers (Invitrogen). One to four colonies generated from each individual tick (at least one male and one female from each cross) were analyzed.

The ITS2 nucleotide sequences generated in this study were deposited in the GenBank database under accession numbers KX130784–KX130800. The entire ITS2 sequences generated from all clones of each tick specimen were manually aligned using GeneDoc software, and a phylogenetic tree was inferred by the maximum parsimony method using PAUP version 4.0b10 with 500 replicates of random addition taxa and tree bisection and reconnection branch swapping; all positions were given equal weight.

### Principal components analysis

A principal components analysis (PCA) based on a Pearson’s correlation matrix was applied on 44 morphometric variables for larvae from both homologous and heterologous crosses to elucidate relationships among the two tick populations. In addition, we included data from the larvae of *O. talaje*, *Ornithodoros rioplatensis* Venzal, Estrada-Peña & Mangold, 2008, and *Ornithodoros guaporensis* Nava, Venzal & Labruna, 2013, all retrieved from Nava et al. [17]. Raw measurements were log (x + 1)-transformed to standardize variances and improve normality.

## Results

**Order Ixodida Leach, 1815**

**Family Argasidae Canestrini, 1890**

**Genus*Ornithodoros*Koch, 1844**

***Ornithodoros rietcorreai*Labruna, Nava & Venzal n. sp.**

***Type-host*:** While all ticks were collected from the environment, they were collected from rocky holes inhabited by *K. rupestris* and bats (Piauí), and in a rocky hill inhabited by *K. rupestris* (Paraíba). These findings suggest that *K. rupestris*, and bats to a lesser extent, serve as hosts for *O. rietcorreai* n. sp., a statement yet to be confirmed.

***Type-locality*:** Jurema Municipality, State of Piauí, Brazil (08°52′53.7″S, 43°09′43.0″W; elevation 557 m).

***Other localities*:** São José de Espinharas Municipality, State of Paraíba, Brazil (06°50’S, 37°24’W; elevation 220 m).

***Type-specimens*:** Holotype: one unfed larva, originated from egg laid in the laboratory by paratype female collected in Jurema Municipality, State of Piauí, Brazil, 22.i.2013, by M. B. Labruna (holotype larva mounted in a slide with other 10 non-paratype larvae). Deposited in the United States National Tick Collection, Statesboro, GA, United States (USNMENT00862200, CEN125635). Paratypes (an asterisk indicates that the specimen was measured): five females*, five males*, collected in rocky hole inhabited by *Kerodon rupestris* and bats (08°52′53.7″S, 43°09′43.0″W; elevation 557 m), Jurema Municipality, State of Piauí, Brazil, 22.i.2013, by M. B. Labruna (CNC-3264, 3265, 3266); five females*, five males*, collected in a rocky hill inhabited by *K. rupestris* (06°50'S, 37°24′W; elevation 220 m), São José de Espinharas Municipality, State of Paraíba, Brazil, 10.ix.2012, by M. B. Labruna, S. Nava, J. M. Venzal (CNC-3267, IBSP-12476, USNMENT00862764, CEN125636); ten unfed larvae*, originated from eggs laid in the laboratory by paratype females collected in São José de Espinharas Municipality, State of Paraíba, as stated above (DPVURU-879); nine unfed larvae* and 45 unfed larvae, originated from eggs laid in the laboratory by paratype females collected in Jurema Municipality, State of Piauí, as stated above (DPVURU-880); 64 unfed larvae, originated from eggs laid in the laboratory by paratype females collected in Jurema Municipality, State of Piauí, as stated above (CNC-3268, INTA-2329, IBSP-12477, USNMENT00862762-00862763, CEN125635); ten males, two females, two nymphs (these nymphs were destroyed for DNA extraction) collected in São José de Espinharas Municipality, State of Paraíba, as stated above (CNC-3273); five males, three females, ten nymphs (two of these nymphs were destroyed for DNA extraction) collected in Jurema Municipality, State of Piauí, as stated above (CNC-3270); 74 unfed larvae, originated from eggs laid in the laboratory by paratype females collected in São José de Espinharas Municipality, State of Paraíba, as stated above (CNC-3271); 32 unfed larvae, originated from eggs laid in the laboratory by paratype females collected in Jurema Municipality, State of Piauí, as stated above (CNC-3269); five males, one female, four nymphs (two of these nymphs were destroyed for DNA extraction) collected in a rocky hole inhabited by *K. rupestris* and bats (08°53′27.4"S, 43°07′53.4"W; elevation 554 m), Jurema Municipality, State of Piauí, Brazil, 23.i.2013, by M. B. Labruna (CNC-3272).

***Representative DNA sequences*:** Mitochondrial 16S rRNA gene, partial sequences: GenBank accession numbers KX130781–KX130782; second internal transcribed spacer (ITS2) of the nuclear ribosomal DNA, complete sequences: GenBank accession numbers KX130784– KX130800.

***ZooBank registration*:** Details of the new species have been submitted to ZooBank (http://zoobank.org/). The Life Science Identifier (LSID) of the article is urn:lsid:zoobank.org:act:106221A8-3781-48DA-B63C-98F4D91BFAAD. The LSID for the new name *Ornithodoros rietcorreai* is urn:lsid:zoobank.org:act:8A6D9021-22D4-447C-A9BA-85FADED53A9C.

***Etymology*:** The species is named for Professor Franklin Riet-Correa, veterinary pathologist, for his outstanding contributions to the scientific development (including parasitology) in the Caatinga biome, where this new species was discovered.

### Description

**Fig. 1:**
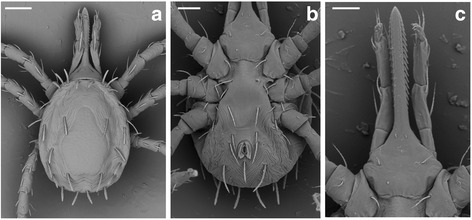
Scanning electron micrographs of *Ornithodoros rietcorreai* n. sp. Larva. **a** Unengorged specimen, dorsal view. **b** Unengorged specimen, ventral view. **c** Ventral capitulum. *Scale-bars*: **a**, **b**, 100 μm; **c**, 50 μm

**Fig. 2:**
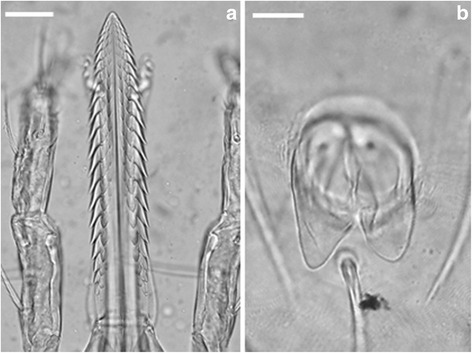
Light microscopy photomicrographs of *Ornithodoros rietcorreai* n. sp. Larva. **a** Hypostome. **b** Anal valves. *Scale-bars*: 25 μm

**Fig. 3:**
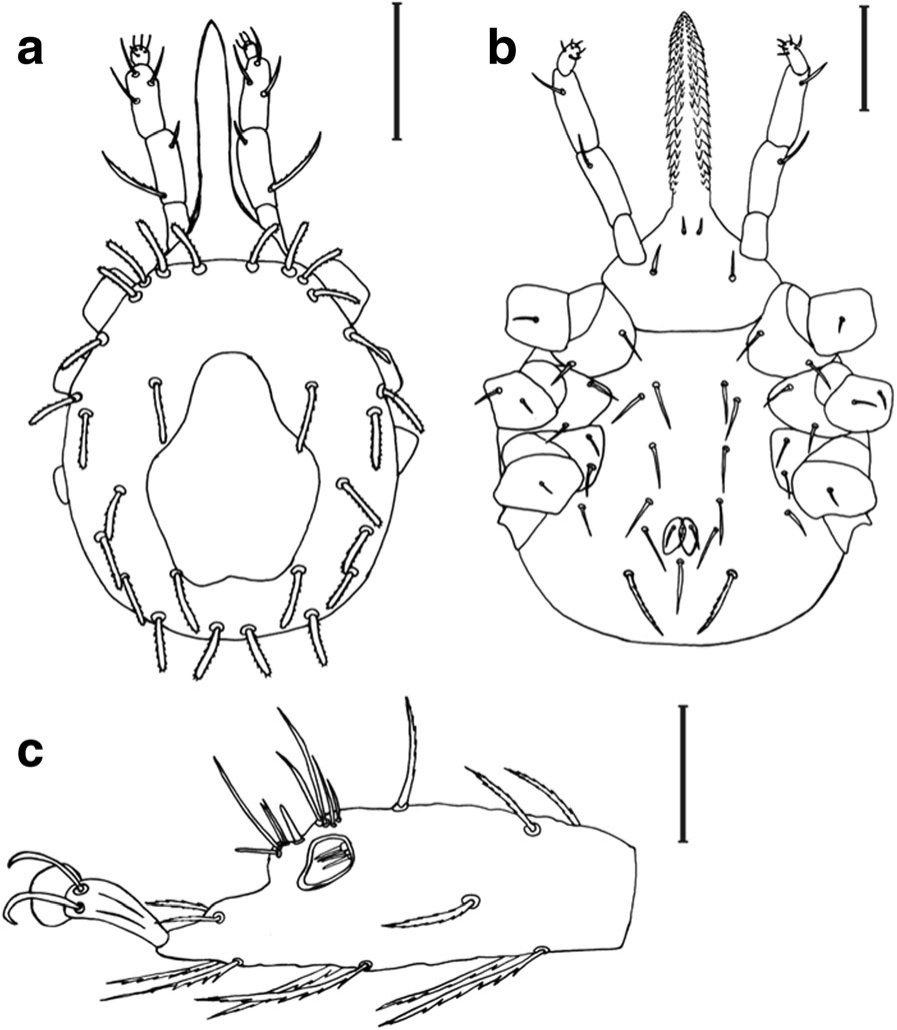
Line drawing of *Ornithodoros rietcorreai* n. sp. Larva. **a** Dorsal view. **b** Ventral view. **c** Tarsus I. Scale-bars: **a**, **b**, 150 μm; **c**, 50 μm

***Larva.*** [Figs. 1, 2 and 3; measurements based on the holotype and 9 paratypes from Piauí (PI-1 ticks) and 10 paratypes from Paraíba (PB ticks).]

Body: Idiosoma oval. Length including capitulum 0.723–0.841 (0.777), length excluding capitulum 0.396–0.475 (0.448), width 0.372–0.465 (0.403). Dorsum: Dorsal plate pyriform, posterior margin slightly concave, length 0.256–0.305 (0.275), width 0.151–0.219 (0.182).

Dorsal surface provided with 14 pairs of setae, seven anterolateral, three central and four posterolateral. Anterolateral setae (Al): Al_1_ length 0.061–0.078 (0.069), Al_2_ length 0.061–0.075 (0.068), Al_3_ length 0.061–0.078 (0.067), Al_4_ length 0.058–0.078 (0.066), Al_5_ length 0.056–0.073 (0.067), Al_6_ length 0.063–0.080 (0.071), Al_7_ length 0.073–0.085 (0.079). Central setae (C): C_1_ length 0.063–0.090 (0.078), C_2_ length 0.068–0.075 (0.071), C_3_ length 0.073–0.088 (0.079). Posterolateral setae (Pl): Pl_1_ length 0.063–0.078 (0.072), Pl_2_ length 0.063–0.081 (0.071), Pl_3_ length 0.058–0.083 (0.069), Pl_4_ length 0.061–0.075 (0.068).

Venter: Ventral surface provided with seven pairs of setae plus 1 pair on anal valves, one posteromedian seta present. Anal valves with long and pointed leaf-shaped ends. Three pairs of sternal setae (St): St_1_ length 0.051–0.073 (0.063), St_2_ length 0.051–0.068 (0.061), St_3_ length 0.056–0.075 (0.065); three pairs of circumanal setae (Ca): Ca_1_ length 0.053–0.071 (0.058), Ca_2_ length 0.058–0.075 (0.069), Ca_3_ length 0.073–0.085 (0.078); posteromedian setae (PM) length 0.053–0.073 (0.063), postcoxal setae (Pc) length 0.041–0.051 (0.045).

Capitulum: Basis capituli hexagonal, posterior margin straight. Length from posterior margin of basis capituli to posthypostomal setae: Ph_1_ 0.129–0.149 (0.138), length from posterior margin of basis capituli to insertion of hypostome 0.154–0.173 (0.163), length from posterior margin of basis capituli to apex of hypostome 0.362–0.406 (0.385), width 0.185–0.210 (0.197). Two pairs of posthypostomal setae; Ph_1_ length 0.019–0.024 (0.021), Ph_2_ length 0.039–0.056 (0.046), distance between Ph_1_ setae 0.019–0.024 (0.023), distance between Ph_2_ setae 0.071–0.083 (0.075). Palpal total length 0.264–0.294 (0.277), segmental length/width from I-IV: (I) 0.049–0.061 (0.057)/0.027–0.039 (0.030), (II) 0.083–0.100 (0.088)/0.034–0.044 (0.037), (III) 0.088–0.107 (0.099)/0.024–0.034 (0.030), (IV) 0.036–0.044 (0.040)/0.019–0.024 (0.021). Setal number on palpal articles I-IV: (I) 0, (II) 4, (III) 5, (IV) 9. Hypostome: length from Ph_1_ to apex 0.232–0.256 (0.248), length from insertion of hypostome in basis capituli to apex 0.207–0.236 (0.225), length to inferior toothed portion to apex 0.190–0.212 (0.204), width in medial basis portion of hypostome 0.044–0.056 (0.049), width in basis portion of hypostome 0.039–0.051 (0.044), pointed apically. Dental formula 3/3 in the anterior half, 2/2 posteriorly almost to base. Dentition: denticles absent from a part at the base. File one with 20 to 21 denticles (typically 21), file two with 20 to 21 (typically 21), and file three with 12 to 15 (typically 14).

Legs: Tarsus I length 0.158–0.202 (0.181), tarsus I width 0.049–0.068 (0.060). Setal formula of tarsus I: one pair apical (A), one distomedian (DM), five paracapsular (PC), one posteromedian (PM), one pair basal (B), one pair apicoventral (AV), one pair midventral (MV), one pair basiventral (BV), and one pair posterolateral (PL). Capsule of Haller’s organ without reticulations.

**Fig. 4:**
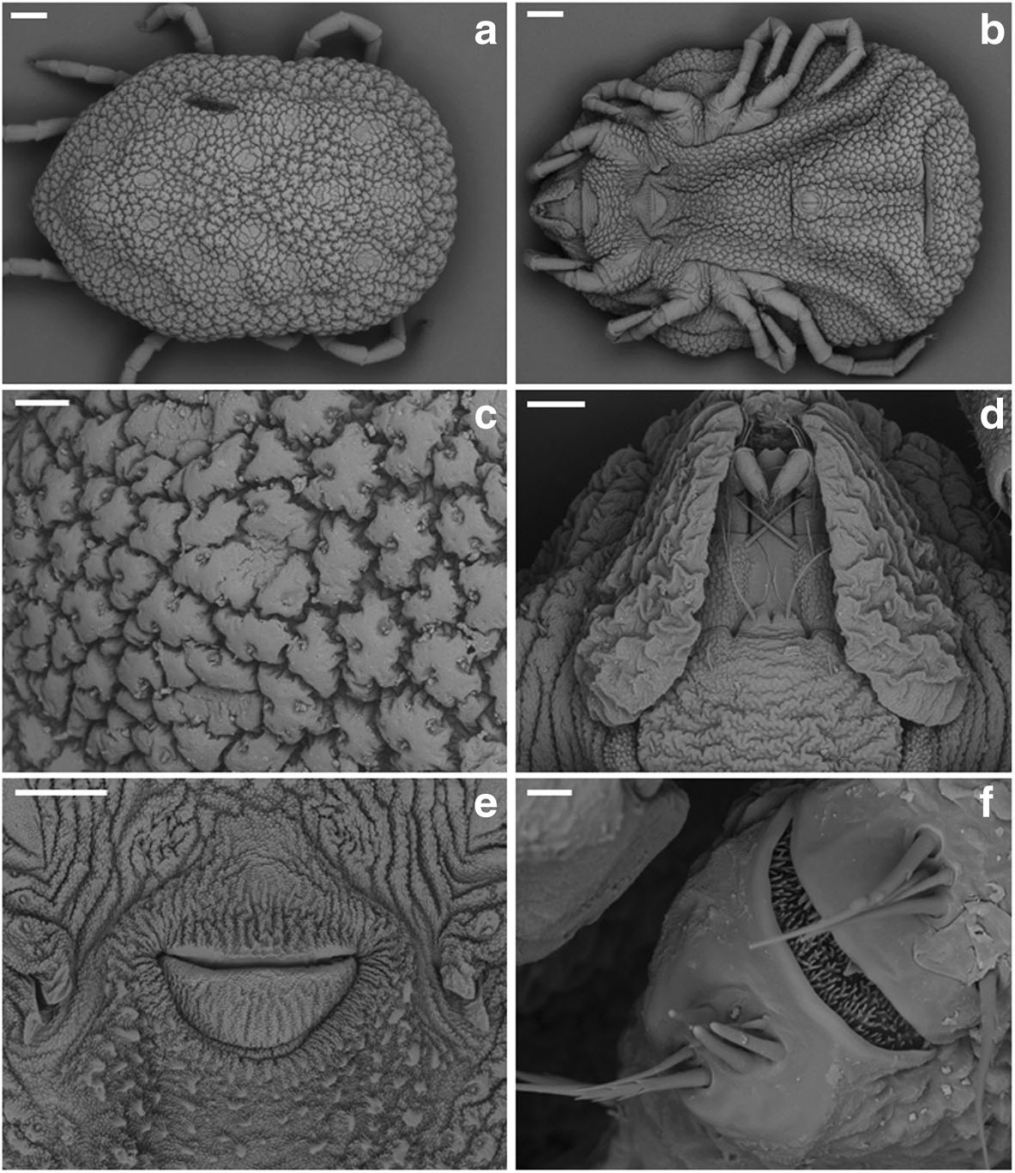
Scanning electron micrographs of *Ornithodoros rietcorreai* n. sp. Female. **a** Dorsal idiosoma. **b** Ventral idiosoma. **c** Detail on dorsal integument. **d** Ventral capitulum. **e** Genital opening. **f** Haller’s organ. *Scale-bars*: **a**, **b**, 400 μm; **c**, **d**, 100 μm; **e**, 200 μm; **f**, 10 μm

***Female.*** [Fig. 4; measurements based on five paratypes from Piauí (PI-1 ticks) and five paratypes from Paraíba (PB ticks).]

Dorsal: Body outline elliptical, pointed anteriorly, curve of posterior border slightly flattened, sides nearly parallel. Length from pointed anterior end to posterior body margin 5.145–6.639 (5.691), breadth 3.254–4.618 (3.730). Submarginal grooves distinct, fused anteriorly. Mammillae large, crowded, irregular in shape, slightly elevated, with short hairs, usually one or two hairs per mammilla. Distinct discs, covered by flattened mammillae in slightly depressed areas over medium area within marginal grooves present.

Ventral: Mammillae as dorsally. Genital opening semi-lunar in shape at level of coxa I, reaching level of the anterior margin of coxa II. Preanal and transverse postanal grooves present; medium postanal groove indicated by depression terminating at transverse postanal groove; coxal folds extending from space between coxae I and II to near level of anus, where they diverge to postero-lateral angles. Anal plate nearly elliptical. Spiracular plate above coxa IV. Small rounded hood; camerostome indistinct; cheeks well developed, with irregular wrinkles, covering mouth parts partially or almost completely.

Capitulum: Basis capituli slightly wider than long, with irregular transverse wrinkles and micromammillated. Palpi moderate in size, with various long setae; article one micromammillated with ventro-medial integumental ridge-like extension covering part of hypostomal base. Hypostome rounded apically with a central notch, reaching to level of articulation of palpal articles three and four. Hypostomal dentition 2/2 with 6–8 denticles per row; toothed portion starting at level of articulation of palpal articles one and two.

Legs: Long, with micromammillated surface; coxae I-II with various mammillae; coxae III-IV with few mammillae over micromammillated surface. Small setae sparsely distributed through articles. Coxa I well separated by coxa II by an inter-coxal fold, coxae II-IV contiguous. Tarsi I-IV with a discrete subapical dorsal protuberance, dorsal humps absent; stout claws and small pulvilli present. Anterior tip of Haller’s organ with distinct anterior and posterior sections; capsule opening transverse, slitlike.

**Fig. 5:**
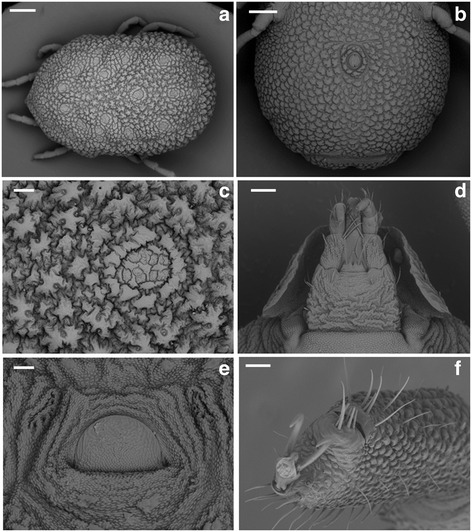
Scanning electron micrographs of *Ornithodoros rietcorreai* n. sp. Male. **a** Dorsal idiosoma. **b** Posterior part of ventral idiosoma. **c** Detail on dorsal integument. **d** Ventral capitulum. **e** Genital opening. **f** Tarsus I showing Haller’s organ. *Scale-bars*: **a**, 600 μm; **b**, 400 μm; **c**, **d**, 100 μm; **e**, 60 μm; **f**, 30 μm

***Male.*** [Fig. 5; measurements based on five paratypes from Piauí (PI-1 ticks) and five paratypes from Paraíba (PB ticks)]

Dorsal: Length from pointed anterior end to posterior body margin 4.110–5.298 (4.659), breadth 3.235–3.565 (3.052). Other features as in female.

Ventral: Genital opening with a flap in the form of half circle, located at level of coxa I. Other features as in females.

Capitulum: As in females.

Legs: As in females.

### Distribution

The geographical distribution of *O. rietcorreai* is restricted to the Caatinga biome in northeastern Brazil, where this tick species was found in two municipalities (in Paraíba and Piauí states), at a distance of ≈ 670 km from each other. Since populations of *K. rupestris* are widespread in the Caatinga biome [1], it is possible that the geographical distribution of *O. rietcorreai* encompasses additional areas within this biome.

### Remarks

*Ornithodoros rietcorreai* n. sp. belongs to the subgenus *Alectorobius* Pocock, 1907 because adults possess cheeks, a typical character for this subgenus, and larvae possess the most typical characters (not exclusive) for the subgenus *Alectorobius*: dorsal plate pyriform, dorsal body surface with 11 to 18 pairs of dorsolateral setae, usually barbed, ventral body surface with 8 or 9 pairs of setae plus a posteromedian seta, and hypostome pointed with dentition 3/3 to 5/5 anteriorly, 2/2 posteriorly to base [18]. Differentiation of species of *Alectorobius* by the features of the adult stage is complicated in many cases [7–9]; therefore the larval stage is indicated for species identification through morphological analysis. The larva of *O. rietcorreai* is distinct from the previously described *Ornithodoros* spp. due to the combination of the following characters: 14 pairs of dorsal setae (7 anterolateral, 3 central and 4 posterolateral pairs), dorsal plate pyriform (length *c.*0.275 mm), hypostome with pointed apex and dental formula 3/3 in the anterior half, 2/2 posteriorly almost to base, and anal valves with long and pointed leaf-shaped ends. On the other hand, the larva of *O. rietcorreai* n. sp. is similar to the species included in the *O. talaje* species group. The larva in this group is characterised by the presence of a pyriform dorsal plate, pointed hypostome, and 17–20 pairs of dorsal setae [19, 20]. Contrastingly, *O. rietcorreai* larvae have 14 pairs of dorsal setae. Other Neotropical species of *Ornithodoros* with 14 pairs of dorsal setae in the larval stage are *Ornithodoros dyeri* Cooley & Kohls, 1940, *Ornithodoros fonsecai* (Labruna & Venzal, 2009), *Ornithodoros knoxjonesi* Jones & Clifford, 1972, *Ornithodoros rossi* Kohls, Sonenshine & Clifford, 1965, *Ornithodoros peropteryx* Kohls, Clifford & Jones, 1969, *Ornithodoros peruvianus* Kohls, Clifford & Jones, 1969, *Ornithodoros yumatensis* Cooley & Kohls, 1941, and *Ornithodoros yunkeri* Keirans, Clifford & Hoogstraal, 1984. Larvae of *O. dyeri*, *O. rossi* and *O. yunkeri* can be differentiated from *O. rietcorreai* by the presence of four pairs of circumanal setae on the ventral idiosoma in the former two species, and the absence of the posteromedian seta in *O. yunkeri*. Larvae of *O. knoxjonesi* and *O. peropteryx* possess a constriction near the hypostome mid-length, which is absent in the hypostome of *O. rietcorreai*.

Larvae of *O. fonsecai* are clearly larger (mean body length 1.020 mm; range: 0.990–1.050 mm) than the larvae of *O. rietcorreai* (mean 0.777 mm; range: 0.723–0.841 mm). *Ornithodoros peruvianus* and *O. yumatensis* differ from *O. rietcorreai* by their larger dorsal plate (mean ˃ 0.310 *vs* 0.275 mm). Additionally, *O. peruvianus* possess fringed setae in tarsus I, and the capsule of Haller’s organ is reticulated in *O. yumatensis*. Finally, *O. rietcorreai* larvae have anal valves with long and pointed leaf-shaped ends, a feature not present in any of the above mentioned species.

**Fig. 6:**
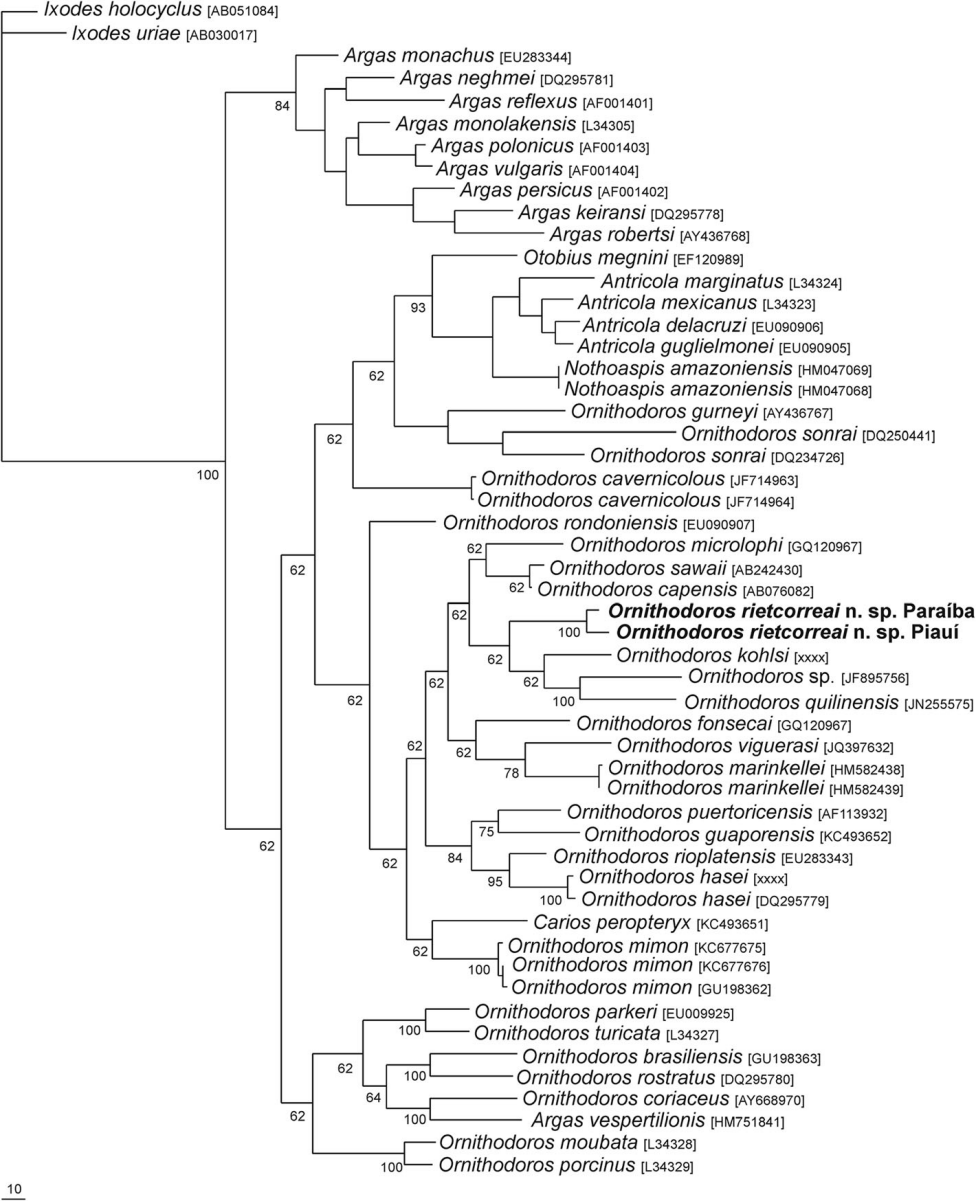
Maximum parsimony (MP) phylogenetic tree based on partial 16S rDNA sequences for *Ornithodoros rietcorreai* n. sp. and other argasid tick species. *Ixodes uriae* and *I. holocyclus* were used as the outgroup. Numbers at nodes represent support values derived from bootstrap (500 replicates)

While *O. riecorreai* is phylogenetically closely related to *Ornithodoros quilinensis* Venzal, Nava & Mangold, 2012, *Ornithodoros xerophylus* Venzal, Mangold & Nava, 2015 and *Ornithodoros kohlsi* Gugliemone & Keirans, 2002 (Fig. 6), the larva of the later species has 19 pairs of dorsal setae (in contrast to 14 in *O. rietocorreai*), and the larvae of both *O. quilinensis* and *O. xerophylus* possess a blunt hypostome, in contrast to the pointed hypostome in *O. rietcorreai* n. sp*.*

### Molecular and phylogenetic analyses

A fragment of the 16S rDNA mitochondrial gene was generated from two nymphs from each of the three localities (PB, PI-1, and PI-2). The two PB nymphs generated an identical haplotype of 426 nucleotides (KX130781), whereas the four PI nymphs (2 PI-I and 2 PI-2) generated an identical haplotype of 427 nucleotides (KX130782). These two distinct haplotypes were 96.7 % (413/427) similar to each other. By BLAST analyses, these haplotypes were most similar (88–89 %) to the sequences available on GenBank for *Ornithodoros capensis* Neumann, 1901 (e.g. JQ824304, AB242431, AB075953) and *Ornithodoros sawaii* Kitaoka & Suzuki, 1973 (e.g. KP899267, KT372792, KP730692). Phylogenetic relationships inferred by analysis of partial sequences of the mitochondrial 16S rDNA gene (Fig. 6) placed the two haplotypes of *O. rietcorreai* n. sp. together and basal to the sequences of *O. kohlsi* from Bolivia, *O. quilinensis* from Argentina, *Ornithodoros* sp. from Bolivia and *O. xerophylus* from Argentina. This clade was sister to *Ornithodoros lahillei* Venzal, González-Acuña & Nava, 2015 from Chile, and then to *O. capensis*, *O. sawaii* and *Ornithodoros microplophi* Venzal, Nava & González-Acuña, 2013.

### Biological analyses and cross-mating assays

Larvae of *O. rietcorreai* fed for 4 to 7 days on *C. callosus* hosts, with the majority (≈80 %) of engorged larvae detaching on the 5th feeding day. At 25 °C and relative humidity (RH) of 80 %, engorged larvae took 5–7 days to molt to N1 nymphs, which molted to N2 nymphs (without feeding) in 10–12 days. These N2 nymphs fed on hamsters in less than 30 min, and then took 21–23 days to molt to N3 nymphs. These later nymphs fed on hamsters in less than 30 min, and took 20–25 days to molt to N4 nymphs, adult males, or adult females. These adult ticks were used for the cross-mating assays.

Results from the crosses with the *O. rietcorreai* colonies from Paraiba State (PB) and Piauí State (PI) are shown in Table 1. After feeding for up to 60 min on hamsters, F_1_ males and females were sorted in couples (♂PI × ♀PI; ♂PB × ♀PB; ♂PB × ♀PI; ♂PI × ♀PB), each couple within a plastic vial. Females from each single couple of either homologous or heterologous crosses produced viable offspring, as numerous highly motile larvae (they were not counted) appeared in each vial between 30 to 60 days after female feeding. The hatched larvae (F_2_ ticks) were reared until the adult stage, which also produced viable offspring (F_3_ larvae). None of the F_1_ female ticks was able to oviposit without previous contact with males, indicating lack of parthenogenesis in this tick species.

**Table 1:** Results of cross-mating experiments of F_1_ adult ticks of two laboratory colonies of *Ornithodoros rietcorreai* n. sp., one from the state of Piauí (PI), and one from the State of Paraíba (PB), Brazil

Cross (♂ × ♀)	No. of F_1_ adult pairs	No. of F_1_ females that oviposited (%)	No. of females that generated F_2_ larvae	Fertility of F_2_ adults
PI × PI	4	4 (100)	4 (100)	Yes^a^
PB × PB	3	3 (100)	3 (100)	Yes^a^
PB × PI	4	4 (100)	4 (100)	Yes^a^
PI × PB	3	3 (100)	3 (100)	Yes^a^
__ × PB^b^	3	0 (0)	0 (0)	–
__ × PI^b^	3	0 (0)	0 (0)	–

^a^Five male and five female F_2_ adults from each F_1_ cross were left together in a vial after a blood meal; numerous larvae were born in each vial several weeks later, confirming fertility of F_2_ adults

^b^These groups consisted of only females with no contact with males, with the purpose to verify if female ticks could reproduce by parthenogenesis after a blood meal

### Molecular analyses of cross-mating ticks

ITS2 sequences were generated from male and female ticks of each of the four cross-mating groups. Regarding ticks derived from the homologous crosses (purebred), three distinct haplotypes (A, B, C) were generated from the ♂PI × ♀PI ticks, and seven distinct haplotypes (D, E, F, G, H, I, J) were generated from the ♂PB × ♀PB ticks. No haplotype was shared by the two homologous crosses. Among ticks derived from the ♂PB × ♀PI heterologous crosses (hybrids), five haplotypes were generated, three unique (K, M, L) and two (E, H) also found in the homologous ♂PB × ♀PB ticks. Among ticks derived from the ♂PI × ♀PB heterologous crosses (hybrids), six haplotypes were generated, four unique (N, O, P, Q) and two (E, H) also found in the homologous ♂PB × ♀PB ticks (Table 2). Generally, the same individual tick contained two or more different haplotypes.

**Table 2:** Number of clones with respective ITS2 haplotypes generated from purebred (from homologous crosses) or hybrid (from heterologous crosses) male and female F_2_ ticks of two populations of *Ornithodoros rietcorreai* n. sp.

Cross (♂ × ♀)	Sex	Clone	ITS2 Haplotype		
			Code	Size (nt)	GenBank accession no.
PI × PI	Male	1	A	436	KX130784
		2	B	437	KX130785
	Female	1	C	428	KX130786
PB × PB	Male	1	D	429	KX130787
		2	E	429	KX130788
		3	G	428	KX130790
	Female 1	1	E	429	KX130788
		2	I	435	KX130792
		3	G	428	KX130790
	Female 2	1	J	435	KX130793
		2	H	430	KX130791
		3	F	429	KX130789
		4	E	429	KX130788
PB × PI	Male	1	H	430	KX130791
	Female	1	E	429	KX130788
		2	M	434	KX130796
		3	K	435	KX130794
		4	L	430	KX130795
PI × PB	Male	1	N	428	KX130797
		2	E	429	KX130788
		3	H	430	KX130791
	Female	1	P	428	KX130799
		2	O	428	KX130798
		3	Q	429	KX130800

The complete ITS2 sequences (428–437 nucleotides), representing 17 haplotypes of 24 clones, were generated from nine specimens of *O. rietcorreai* (Table 2). Absolute divergence values between the ITS2 sequences are shown in Table 3. Differences between haplotypes A, B, C (homologous cross ♂PI × ♀PI) varied from 0.2 to 7.1 %; differences between haplotypes D, E, F, G, H, I, J (homologous cross ♂PB × ♀PB) varied from 0.2 to 3.5 %; differences between haplotypes K, L, M (heterologous cross ♂PB × ♀PI) varied from 1.4 to 4.1 %; and differences between haplotypes N, O, P, Q (heterologous cross ♂PI × ♀PB) varied from 0.2 to 3.5 % (Table 3). Comparing the four cross-mating groups to each other, haplotypes A-C (♂PI × ♀PI) differed by 1.4–7.6 % from haplotypes D-J (♂PB × ♀PB), whereas haplotypes K-M (♂PB × ♀PI) differed by 0.2–8.5 % from haplotypes N-Q (♂PI × ♀PB). In addition, haplotypes A-C (♂PI × ♀PI) differed by 0.7–7.1 % from K-M (♂PB × ♀PI), and by 0.7–8.5 % from N-Q (♂PI × ♀PB), whereas haplotypes D-J (♂PB × ♀PB) differed by 0.2–4.1 % from K-M (♂PB × ♀PI), and 2.3–8.9 % from N-Q (♂PI × ♀PB).

**Table 3:** Absolute divergence matrix (%) between ITS2 sequences (428 to 437-bp) of *Ornithodoros rietcorreai* n. sp. from four cross-mating groups: ♂PI × ♀PI ticks (haplotypes A-C); ♂PB × ♀PB ticks (haplotypes D-J); ♂PB × ♀PI ticks (haplotypes E, H, K-M); and ♂PI × ♀PB ticks (haplotypes E, H, N-Q)

HC	A	B	C	D	E	F	G	H	I	J	K	L	M	N	O	P	Q
A	–																
B	0.2	–															
C	6.9	7.1	–														
D	2.8	3.0	4.7	–													
E	2.5	2.8	4.4	0.2	–												
F	2.8	3.0	4.7	0.5	0.2	–											
G	2.8	3.0	4.2	0.5	0.2	0.5	–										
H	2.3	2.5	4.7	0.5	0.2	0.5	0.5	–									
I	1.6	1.8	7.6	3.5	3.2	3.5	3.5	3.4	–								
J	1.4	1.6	7.4	3.2	3.0	3.2	3.2	3.2	0.2	–							
K	0.7	0.9	6.2	2.1	1.8	2.1	2.1	2.1	1.4	1.2	–						
L	3.0	3.2	5.4	1.2	0.9	1.2	1.2	0.7	4.1	3.9	2.8	–					
M	1.6	1.8	7.1	3.5	3.2	3.5	3.0	3.4	0.5	0.2	1.4	4.1	–				
N	5.1	5.3	2.1	2.8	2.6	2.8	2.3	2.8	5.8	5.5	4.4	3.5	5.3	–			
O	5.3	5.5	2.3	3.0	2.8	3.0	2.6	3.0	6.0	5.8	4.6	3.7	5.5	0.2	–		
P	6.9	7.1	0.7	4.7	4.4	4.7	4.2	4.7	7.6	7.4	6.2	5.4	7.1	1.9	2.1	–	
Q	8.2	8.5	1.6	6.1	5.8	6.1	5.6	6.0	8.9	8.7	7.6	6.7	8.5	3.3	3.5	1.4	–

*Abbreviation*: *HC* ITS2 haplotype code (see Table 2)

**Fig. 7:**
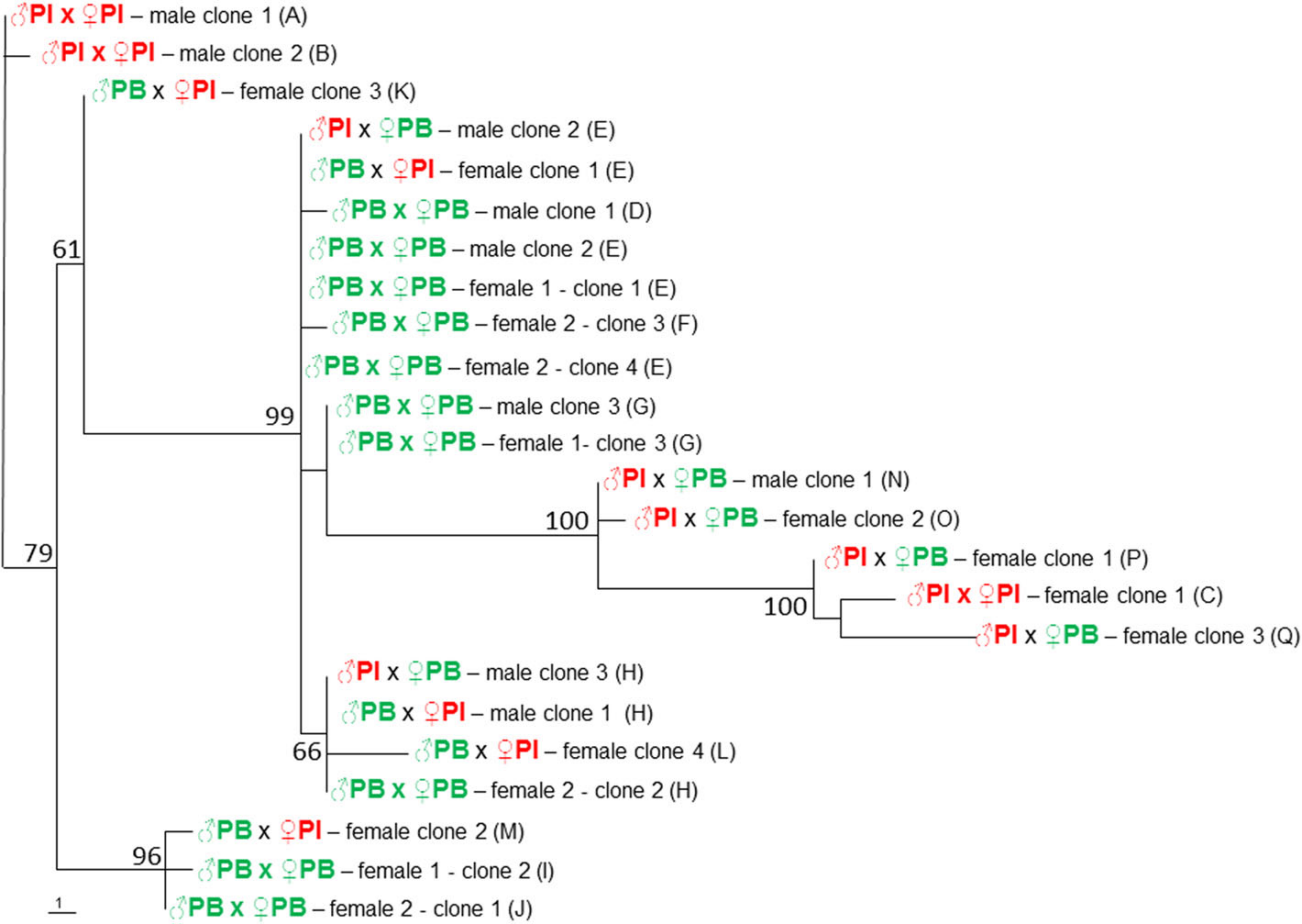
Maximum parsimony (MP) phylogenetic tree based on complete ITS2 sequences for *Ornithodoros rietcorreai* n. sp. adult ticks (males and females) derived from cross-mating experiments of two tick populations, one from the State of Piauí (PI) and one from the State of Paraíba (PB). Numbers at nodes are support values derived from bootstrap (500 replicates). Capital letters within parentheses indicate ITS2 haplotype codes (see Table 2)

A phylogenetic tree inferred by the ITS2 sequences generated in this study (Fig. 7) segregated the ticks of the homologous crosses into distinct clades; i.e. ♂PI × ♀PI ticks did not share any clade with ♂PB × ♀PB ticks. On the other hand, sequences from heterologous crosses (♂PI × ♀PB or ♂PB × ♀PI) grouped within clades containing sequences from homologous crosses (♂PI × ♀PI or ♂PB × ♀PB ticks).

### Morphological analysis of cross-mating ticks and principal components analysis

**Fig. 8:**
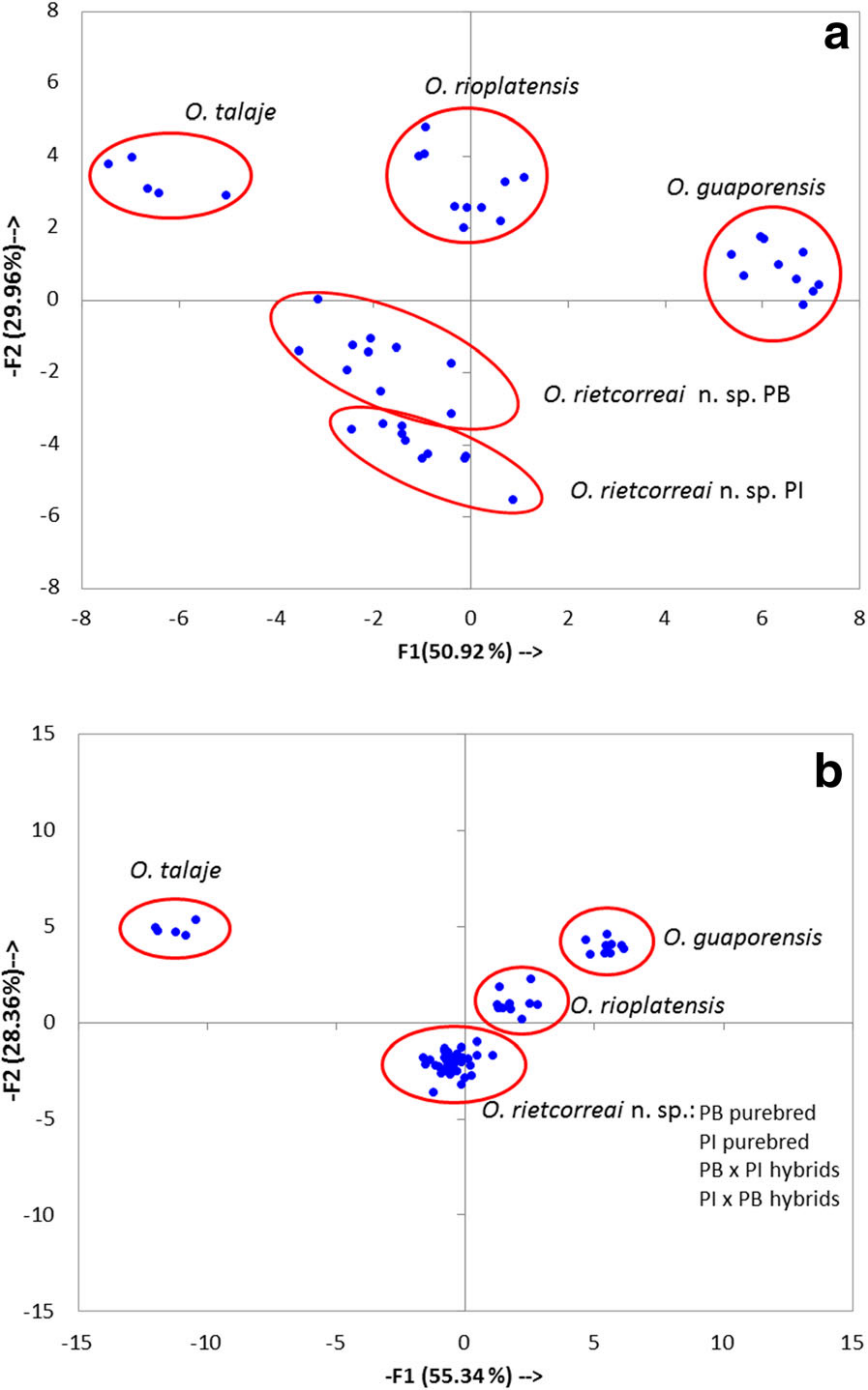
Principal components analysis (PCA) of the body and setal measurements of the larvae of *Ornithodoros rietcorreai* n. sp., *Ornithodoros guaporensis*, *Ornithodoros rioplatensis* and *Ornithodoros talaje*, using the features detailed in Table 4. Morphometric variables for the larvae of *O. guaporensis*, *O. rioplatensis* and *O. talaje* were retrieved from Nava et al. [17]. Each point represents the position of a measured specimen in the reduced morphometric space. **a** Analysis including *O. rietcorreai* purebred larvae from homologous crosses of Paraiba (PB) and of Piauí (PI). **b** Analysis including *O. rietcorreai* purebred larvae from homologous crosses of Paraíba (PB) and Piauí (PI), and hybrid larvae from heterologous crosses (♂PB × ♀PI; ♂PI × ♀PB)

Larvae produced by adult ticks of all four crosses (♂PB × ♀PB; ♂PI × ♀PI; ♂PB × ♀PI; ♂PI × ♀PB) were used for morphological analysis through PCA. Two PCA analyses were carried out with larval morphometric characters (Table 4, Fig. 8). One PCA analysis included purebred ticks from two different populations, Paraíba and Piauí (Fig. 8a), and a second PCA analysis was performed with purebred and hybrid ticks (Fig. 8b). Both PCA analyses showed a clear separation between *O. guaporensis*, *O. rioplatensis*, *O. talaje* and *O. rietcorreai*. In the PCA analysis that included *O. rietcorreai* larvae only from homologous crosses, the first principal component (explaining 50.92 % of the total variance) was mainly loaded by the variables body length, palpal length, length of hypostome and circumanal setae, and the second component (explaining 29.96 % of the total variance) was principally loaded by the length of basis capituli and dorsal setae (total and dorsolateral). In this analysis, there was a clear separation of the two purebred populations of *O. rietcorreai*, although in a much narrower magnitude than the separation observed between the four tick species (Fig. 8a). In the PCA analysis constructed with purebred (homologous crosses) and hybrid (heterologous crosses) ticks, the first principal component (explaining 55.34 % of the total variance) was mainly loaded by the variables body length (a), pairs of dorsal setae (central), length of basis capituli, length of hypostome and circumanal setae, while the second component (explaining 28.36 % of the total variance) was loaded most heavily by dorsal setae (total and dorsolateral), palpal length (article IV) and hypostome width. In this second analysis, there was no clear separation between the four populations of *O. rietcorreai,* although they were clearly separated from the other three species of *Ornithodoros* (Fig. 8b).

**Table 4:** Morphometric variables (range and mean values in millimeters) of *Ornithodoros rietcorreai* larvae from Paraíba (♂PB × ♀PB homologous cross; *n* = 10); Piauí (♂PI × ♀PI homologous cross; *n* = 10); Paraíba × Piauí (♂PB × ♀PI heterologous cross; *n* = 10); and Piauí × Paraíba (♂PI × ♀PB heterologous cross; *n* = 7) utilized in the principal components analysis (PCA)

	*O. rietcorreai* Paraíba	*O. rietcorreai* Piauí	*O. rietcorreai* Paraíba × Piauí	*O. rietcorreai* Piauí × Paraíba
Body length^a^	0.723–0.784 (0.758)	0.742–0.841 (0.797)	0.732–0.762 (0.748)	0.723–0.782 (0.754)
Body length^b^	0.396–0.465 (0.439)	0.435–0.475 (0.457)	0.416–0.445 (0.435)	0.435–0.475 (0.456)
Body width	0.372–0.411 (0.393)	0.386–0.465 (0.414)	0.346–0.416 (0.381)	0.366–0.416 (0.393)
Dorsal plate: length	0.256–0.293 (0.269)	0.268–0.305 (0.281)	0.254–0.273 (0.263)	0.256–0.278 (0.264)
Dorsal plate: width	0.151–0.188 (0.167)	0.185–0.219 (0.197)	0.163–0.195 (0.179)	0.166–0.195 (0.172)
Dorsal setae pairs: total	14	14	14	14
Dorsal setae pairs: dorsolateral	11	11	11	11
Dorsal setae pairs: central	3	3	3	3
Central setae 1: length	0.063–0.085 (0.076)	0.073–0.090 (0.081)	0.073–0.085 (0.079)	0.078–0.085 (0.082)
Central setae 2: length	0.068–0.075 (0.072)	0.068–0.075 (0.071)	0.068–0.075 (0.071)	0.071–0.085 (0.079)
Central setae 3: length	0.075–0.088 (0.081)	0.063–0.083 (0.078)	0.078–0.093 (0.085)	0.075–0.090 (0.084)
Sternal setae 1: length	0.051–0.063 (0.057)	0.063–0.073 (0.069)	0.056–0.068 (0.061)	0.061–0.068 (0.063)
Sternal setae 2: length	0.051–0.061 (0.057)	0.061–0.068 (0.064)	0.056–0.063 (0.058)	0.054–0.066 (0.061)
Sternal setae 3: length	0.056–0.066 (0.060)	0.066–0.075 (0.070)	0.053–0.066 (0.060)	0.058–0.073 (0.064)
Circumanal setae 1: length	0.053–0.061 (0.056)	0.056–0.071 (0.061)	0.051–0.061 (0.057)	0.051–0.063 (0.056)
Circumanal setae 2: length	0.058–0.073 (0.063)	0.065–0.075 (0.071)	0.058–0.073 (0.065)	0.066–0.073 (0.069)
Circumanal setae 3: length	0.073–0.085 (0.076)	0.078–0.085 (0.082)	0.078–0.083 (0.079)	0.075–0.087 (0.081)
Posteromedian setae: length	0.041–0.049 (0.044)	0.044–0.051 (0.045)	0.036–0.056 (0.042)	0.041–0.048 (0.044)
Postcoxal setae: length	0.053–0.073 (0.063)	0.058–0.071 (0.063)	0.053–0.061 (0.059)	0.053–0.073 (0.063)
Length of basis capituli^c^	0.129–0.134 (0.133)	0.136–0.149 (0.144)	0.112–0.134 (0.126)	0.124–0.142 (0.134)
Length of basis capituli^d^	0.154–0.171 (0.158)	0.158–0.173 (0.169)	0.134–0.158 (0.146)	0.149–0.158 (0.153)
Length of capitulum^e^	0.362–0.396 (0.380)	0.376–0.406 (0.390)	0.362–0.382 (0.376)	0.366–0.386 (0.374)
Width of basis capituli	0.185–0.195 (0.191)	0.197–0.210 (0.204)	0.171–0.190 (0.182)	0.183–0.219 (0.200)
Palpal length	0.264–0.294 (0.278)	0.268–0.288 (0.277)	0.256–0.274 (0.262)	0.267–0.287 (0.276)
Length article I	0.049.0.061 (0.054)	0.058–0.061 (0.059)	0.049–0.061 (0.053)	0.049–0.061 (0.056)
Length article II	0.083–0.100 (0.092)	0.083–0.085 (0.084)	0.085–0.100 (0.094)	0.083–0.097 (0.089)
Length article III	0.088–0.107 (0.100)	0.097–0.100 (0.098)	0.097–0.110 (0.104)	0.098–0.107 (0.100)
Length article IV	0.036–0.044 (0.039)	0.039–0.044 (0.041)	0.039–0.046 (0.041)	0.034–0.044 (0.039)
Width article I	0.027–0.039 (0.030)	0.027–0.029 (0.028)	0.027–0.029 (0.028)	0.027–0.032 (0.028)
Width article II	0.034–0.044 (0.038)	0.034–0.036 (0.035)	0.036–0.039 (0.037)	0.034–0.036 (0.035)
Width article III	0.024–0.032 (0.028)	0.029–0.034 (0.031)	0.027–0.032 (0.029)	0.031–0.034 (0.032)
Width article IV	0.019–0.024 (0.022)	0.019–0.022 (0.020)	0.019–0.022 (0.021)	0.019–0.022 (0.020)
Hypostome: length^f^	0.232–0.254 (0.247)	0.237–0.256 (0.249)	0.236–0.246 (0.240)	0.227–0.241 (0.236)
Hypostome: length^g^	0.190–0.210 (0.202)	0.202–0.212 (0.206)	0.207–0.224 (0.213)	0.188–0.202 (0.196)
Hypostome: width at midlength	0.039–0.046 (0.043)	0.041–0.051 (0.046)	0.043–0.049 (0.044)	0.041–0.046 (0.043)
Hypostome: width at the base	0.044–0.053 (0.049)	0.044–0.056 (0.050)	0.046–0.058 (0.051)	0.040–0.056 (0.050)
Apical dental formula	3	3	3	3
Median dental formula	3	3	3	3
Basal dental formula	2	2	2	2
Denticles in hypostomal row 1	20 to 21	18 to 20	20 to 22	20 to 22
Denticles in hypostomal row 2	20 to 21	18 to 19	19 to 21	20 to 22
Denticles in hypostomal row 3	12 to 15	13 to 14	13 to 16	13 to 15
Tarsus I: length	0.158–0.190 (0.167)	0.190–0.202 (0.195)	0.146–0.158 (0.155)	0.166–0.171 (0.168)
Tarsus I: width	0.049–0.061 (0.056)	0.061–0.068 (0.063)	0.049–0.061 (0.056)	0.058–0.063 (0.061)

^a^Including capitulum

^b^Not including capitulum

^c^Length of basis capituli: measured from the posterior margin of basis capituli to posthypostomal setae 1 (Ph1)

^d^Length of basis capituli: measured from the posterior margin of basis capituli to insertion of hypostome

^e^Length of capitulum: measured from the posterior margin of basis capituli to the anterior end of hypostome

^f^Measured to Ph1

^g^Measured to the posterior end of the toothed portion

## Discussion

The argasid *O. rietcorreai* n. sp. is described from rock cavy habitats in three distinct areas of the Caatinga biome, two in Piauí State, and one in Paraíba State. Among the previous reports of argasid ticks from the rock cavy or its resting places in the Caatinga biome [2–6], at least one [5] was from the same region where we collected ticks in Piauí State. Because it is currently considered that the species *O. talaje* does not occur in Brazil [10], it is possible that at least part of these earlier records from the Caatinga refer to *O. rietcorreai.*

Because there were a few larval morphological differences between Paraíba (PB) and Piuaí (PI) populations of *O. rietcorreai* (Table 4, Fig. 8)*,* associated with 3.3 % divergence between mitochondrial 16S rDNA sequences, we decided to perform cross-mating experiments in order to verify the reproductive compatibility of the two tick populations. Once our results showed that PB and PI ticks were reproductively compatible, we conclude that the morphological and genetic differences observed between Paraíba (PB) and Piuaí (PI) populations are merely intraspecific, excluding the hypothesis that they could represent two different tick species.

Our analysis of ITS2 sequences revealed that this genetic marker is usually represented by distinct haplotypes within the same individual tick, as for example, the presence of four different haplotypes (J, H, F, E) in a single PB-female tick, with divergence values varying from 0.2 to 3.2 % (Tables 2 and 3). Such intragenomic variability of ITS2 sequences, previously reported for *Ixodes* ticks from North America, may preclude the reliability of ITS2 to build phylogenetic species trees [21]. On the other hand, a number of studies with different tick genera have successfully applied ITS2 sequences to build phylogenetic analyses [22–25]. It appears that the heterogeneity of ITS2 vary greatly among different tick genera or even among different tick species of one genus. For instance, a recent study showed that the ixodid species *Amblyomma cajennense* (Fabricius, 1787) was represented by a single ITS2 haplotype among 15 geographically distinct populations, whereas a closely related species, *Amblyomma sculptum* Berlese, 1888, was represented by ten distinct haplotypes from different geographical populations [26]. High levels of ITS2 variability and incomplete homogenization of ITS2 sequences may be caused by introgression associated with hybridization or by incomplete lineage sorting. In this sense, both PCA with purebred ticks and 16S rDNA sequence analyses showed that there is a degree of isolation between Paraíba and Piuaí populations of *O. rietcorreai*, but the results of the cross-mating experiment, the analysis of the ITS2 sequences and the PCA performed with purebred and hybrid ticks clearly indicated that the ticks from both populations belong to the same specific entity.

Although ITS2 might not be a suitable molecular marker for phylogenetic inferences of *O. rietcorreai* (due to great heterogeneity), we constructed a phylogenetic tree with all 17 haplotypes generated in the present study (Fig. 7). In this analysis, the population-specific haplotypes (A-C for PI ticks; D-J for PB ticks) segregated at different clades, suggesting that they could be useful molecular markers for discrimination of the two tick populations. Moreover, the fact that hybrid ticks (from the crosses ♂PI × ♀PB or ♂PB × ♀PI) had some of these haplotypes, and also additional haplotypes that grouped in the phylogenetic tree with either of the two purebred ticks (♂PI × ♀PI or ♂PB × ♀PB), indicate that they are in fact hybrid ticks derived from the crosses of the two tick populations. It must be emphasized that until the completion of the present study, the only ITS2 sequence for argasid species available on GenBank was a 583 bp sequence for *Argas africolumbae* Hoogstraal, Kaiser, Walker, Ledger, Converse & Rice, 1975 (KF984488), which did not align with our *O. rietcorreai* sequences, precluding any analyses of the *O. rietcorreai* ITS2 sequences with other argasid tick species.

## Conclusion

*Ornithodoros rietcorreai* is described as a new species associated with *K. rupestris* in Brazil, increasing the Brazilian tick fauna to 70 species.
